# *Lycium barbarum* polysaccharides protect mice from hyperuricaemia through promoting kidney excretion of uric acid and inhibiting liver xanthine oxidase

**DOI:** 10.1080/13880209.2020.1817951

**Published:** 2020-09-18

**Authors:** Xin Yu, Lu Zhang, Ping Zhang, Jia Zhi, Ruinan Xing, Lianqi He

**Affiliations:** a Department of Nuclear Medicine, General Hospital of Fushun Mining Bureau of Liaoning Health Industry Group, Fushun, Liaoning, China; b Department of Endocrinology, General Hospital of Fushun Mining Bureau of Liaoning Health Industry Group, Fushun, Liaoning, China; c Department of Endocrinology, Central Hospital of Fushun, Fushun, Liaoning, China; d Dermatological Department, General Hospital of Fushun Mining Bureau of Liaoning Health Industry Group, Fushun, Liaoning, China; e Department of Cardiology, Zhongshan Hospital Xiamen University, Xiamen, Fujian, China; f Department of Cardiology, Central Hospital of Fushun, Fushun, Liaoning, China

**Keywords:** Organic anti-transporter 1, organic anti-transporter 3, glucose transporter 9

## Abstract

**Context:**

*Lycium barbarum* L. (Solanaceae) polysaccharides (LBPs) are important active constituents that have demonstrated kidney protection.

**Objective:**

This study investigated the effect of LBPs on hyperuricaemia and explored the underlying mechanism in mice.

**Materials and methods:**

Thirty-six C57BL/6 mice were randomly divided into the control group, hyperuricaemia group, allopurinol group (5 mg/kg) and three LBP groups (n = 6). The LBP groups were treated orally with LBPs at 50, 100 and 200 mg/kg body weight for 7 days. We examined the levels of serum uric acid (S_UA_) and urinary uric acid (U_UA_), as well as xanthine oxidase (XOD) activities. mRNA and protein were quantified by quantitative real-time PCR and Western blotting, respectively.

**Results:**

LBPs treatment (100 and 200 mg/kg) significantly reduced the S_UA_ levels to 4.83 and 4.48 mg/dL, and markedly elevated the U_UA_ levels to 4.68 and 5.18 mg/dL (*p* < 0.05), respectively, while significantly increased the mRNA and protein expression levels of renal organic anti-transporter 1 (OAT1) and organic anti-transporter 3 (OAT3), and markedly decreased the levels of glucose transporter 9 (GLUT9) (*p* < 0.05). Additionally, the serum XOD activities were reduced to 31.5 and 31.1 mU/mL, and the liver XOD activities were reduced to 80.6 and 75.6 mU/mL after treatment with 100 and 200 mg/kg LBPs (*p* < 0.01), respectively.

**Discussion and conclusions:**

This study demonstrated the potential role of LBPs in reducing the uric acid level in hyperuricemic mice. A border study population should be evaluated. These results are valuable for the development of new anti-hyperuricaemia agents from LBPs.

## Introduction

Hyperuricaemia, characterized by high blood uric acid levels, is a metabolic disease. Under the condition of chronic hyperuricaemia, uric acid is crystallized and deposited as monosodium urate in the joint, being closely correlated with gout. Xanthine oxidase (XOD), the key enzyme in purine metabolism in the liver, is responsible for catalysing purine to uric acid (Choi et al. 2005; George and Struthers 2009). Thus, reducing the activity of XOD could be used to treat hyperuricaemia. In addition to the synthesis of uric acid, molecules responsible for the secretion and reabsorption of uric acid in the kidney also contribute to abnormal uric acid levels in the blood, such as renal organic anti-transporter 1 (OAT1), organic anti-transporter 3 (OAT3) and glucose transporter 9 (GLUT9). Presently, two types of agents have been used in the treatment of hyperuricaemia: one mainly accelerates uric-acid metabolism, and the other is used primarily to inhibit the activity of XOD, such as allopurinol. However, these agents have many adverse reactions, such as allergic reactions, kidney dysfunction, gastrointestinal symptoms and fever (Chung et al. 2015; Yang et al. 2015). Therefore, a safer and more effective agent with no side effects is needed for the treatment of hyperuricaemia.

*Lycium barbarum* L. (Solanaceae), a traditional Chinese medicine, has the effects of nourishing the liver and kidney and replenishing vital essence to improve eyesight. *Lycium barbarum* polysaccharides (LBPs) are the main effective ingredient of *Lycium barbarum*, comprised of glucose, arabinose, galactose, mannose, xylose, rhamnose and fucose (Zeng et al. 2019; Liu et al. 2020). Studies have demonstrated that LBPs have multiple pharmacological functions, including antioxidant, anti-inflammatory, antitumor, immune regulation, neuroprotective, hypoglycaemic and hypolipidemic effects (Li XM et al. 2007; Miao et al. 2010; Cheng et al. 2015; Xing et al. 2016; Po et al. 2017; Tang ZY et al. 2017; Pop et al. 2020). Moreover, Li J et al. (2017) demonstrated the protective effects of LBPs in renal damage. Thus far, the use of LBPs to treat hyperuricaemia has not been reported. In this study, we examined the efficacy of LBPs in reducing the serum uric acid levels in a mouse model of hyperuricaemia induced by potassium oxonate. We also investigated the possible mechanisms of this effect.

## Materials and methods

### Chemicals and reagents

LBPs (≥90%) were purchased from Beijing Solarbio Science & Technology Co., Ltd. (Beijing, China). Potassium oxonate (97%) and allopurinol (≥99%) was purchased from Sigma-Aldrich Co. LLC (Shanghai, China). Uric acid (UA), serum creatinine (S_CR_), blood urine nitrogen (BUN) and xanthine oxidase activity assay kit were purchased from Abcam Inc. (Cambridge, UK). The RNA isolation kit was obtained from Promega Biotechnology Co., Ltd. (Beijing, China). PrimeScript^TM^ RT reagent kit with gDNA Eraser and TB Green^®^ Premix Ex Taq™ II (Tli RNaseH Plus) was purchased from Takara Biomedical Technology Co., Ltd. (Dalian, China). Protease and phosphatase inhibitors were purchased from Thermo Fisher Scientific Inc. (Waltham, MA, USA). Anti-OAT1, anti-OAT3 and β-actin antibody were purchased from Santa Cruz Biotechnology, Inc. (Dallas, TX, USA). Anti-GLUT9 was purchased from Abcam Inc. (Cambridge, UK). The Amersham ECL System was purchased from GE Healthcare (Pittsburgh, PA, USA). Other biochemical reagents and chemicals were of analytical grade.

### Animals

Eight-week-old C57BL/6 male mice (Vital River Laboratory Animal Technology Co., Ltd., Beijing, China), weighing 20–25 g, were housed at room temperature (23 ± 1 °C) under standard conditions: humidity of 50 ± 5%, allowing free access to water and food under 12 h light/dark cycle (light at 8:00 am). The study was approved by the Ethics Committee of Central Hospital of Fushun for Animal Experiments and was performed according to the Guide for the Care and Use of Laboratory Animals published by the National Institutes of Health (NIH Publication 86-23, 1985 revision).

Thirty-six mice were randomly divided into the control group, hyperuricaemia group, allopurinol (5 mg/kg) group and LBP groups (50, 100 and 200 mg/kg) (n = 6). The concentrations of LBPs were determined according to the literature (Wu et al. 2010). Potassium oxonate, a urate oxidase inhibitor, was used to induce a hyperuricaemia mouse model (Stavric et al. 1975). In addition to the control group, the remaining five groups were intraperitoneally injected with potassium oxonate (250 mg/kg) at 8:00 am for seven consecutive days. Allopurinol or LBPs (50, 100 and 200 mg/kg) were orally administered at 9:00 am for seven consecutive days in the corresponding group.

Finally, whole blood was collected 1 h after the last administration on the 7^th^ day. After coagulation for 1 h at room temperature, the blood was centrifuged at 4000 *g* for 10 min and the serum was collected. The urine was collected using a mouse metabolic cage. The serum and urine samples were stored at −20 °C until analysis. The liver and kidney tissues of the mice were quickly separated on ice and stored at −80 °C for testing.

### Blood and urine sample analyses

The collected serum and urine samples of the mice were used to detect the levels of serum uric acid (S_UA_), S_CR_, BUN and urinary uric acid (U_UA_) according to the manufacturer’s instructions using appropriate kits.

### Serum and hepatic XOD activity analysis

Briefly, the liver tissue of the mice was thoroughly homogenized in an ice bath by placing it in sodium phosphate buffer at pH 7.4. The homogenate was centrifuged at 4000 *g* for 20 min at 4 °C to extract the supernatant for subsequent use. The serum and hepatic XOD activities were measured using the xanthine oxidase activity assay kit according to the manufacturer’s instructions. The absorbance of each well was measured at 570 nm using an Infinite M Plex Fully Loaded Multimode Plate Reader (Tecan, Männedorf, Switzerland). Each assay was performed in duplicate. XOD activity (mU/mL) was calculated using the formula provided by the manufacturer’s instructions.

### RNA isolation and quantitative real-time PCR

Total RNA was extracted from mouse renal tissue using the RNA isolation kit, and quantitative real-time PCR using PrimeScript^TM^ RT reagent kit with gDNA Eraser and TB Green^®^ Premix Ex Taq™ II (Tli RNaseH Plus) was performed according to our previous report (He et al. 2019) with the following primers: Slc22a6 (OAT1)-F: 5′-TCTGCCTCTCTATGCTGTGG-3′; Slc22a6 (OAT1)-R: 5′-GATTGTATGGCCCCTCGGTA-3′; Slc22a8 (OAT3)-F: 5′-GCCAACCACAACTTGCTACA-3′; Slc22a8 (OAT3)-R: 5′-GATCCAGCCATCCAAGCATG-3′; Slc2a9 (GLUT9)-F: 5′-GGCTCCTTTTCCCTTTCATC-3′; Slc2a9 (GLUT9)-R: 5′-CTGAGCCTTGTTCCTCTTGG-3′; GAPDH-F: 5′-AGTGTTTCCTCGTCCCGTAG-3′; GAPDH-R: 5′-GCCGTGAGTGGAGTCATACT-3′.

The relative mRNA expression levels of Slc22a6, Slc22a8 and Slc2a9 were normalized to that of GAPDH.

### Western blotting

Protein extracts were obtained following the collection of kidney tissue in RIPA lysis buffer supplemented with protease and phosphatase inhibitors. Western blotting was performed as previously described (He et al. 2019). The PVDF membrane was incubated with anti-OAT1, anti-OAT3 or anti-GLUT9 at a dilution of 1:200–1:1000 overnight at 4 °C. An antibody against β-actin (1:1000) was used as a loading control. A horseradish peroxidase-labeled secondary antibody was added for 1 h at room temperature, and the enzymatic substrate was added. The Amersham ECL System was used to develop the blot, and the films were scanned for quantification by densitometry using ImageJ software (National Institutes of Health, Bethesda, MD, http://imagej.nih.gov/ij/, 1997e2012).

### Statistical analysis

All the data in this study were analysed using SPSS 17.0 software for Windows (IBM Corp., Armonk, NY, USA). The data were expressed as the means ± standard deviation (SD). Statistical comparisons between the two groups were performed by Student’s *t*-test. A *p* value <0.05 was considered statistically significant.

## Results

### Effect of LBPs on the S_UA_, S_CR_, BUN and U_UA_ levels in mice with hyperuricaemia

**Figure 1.**
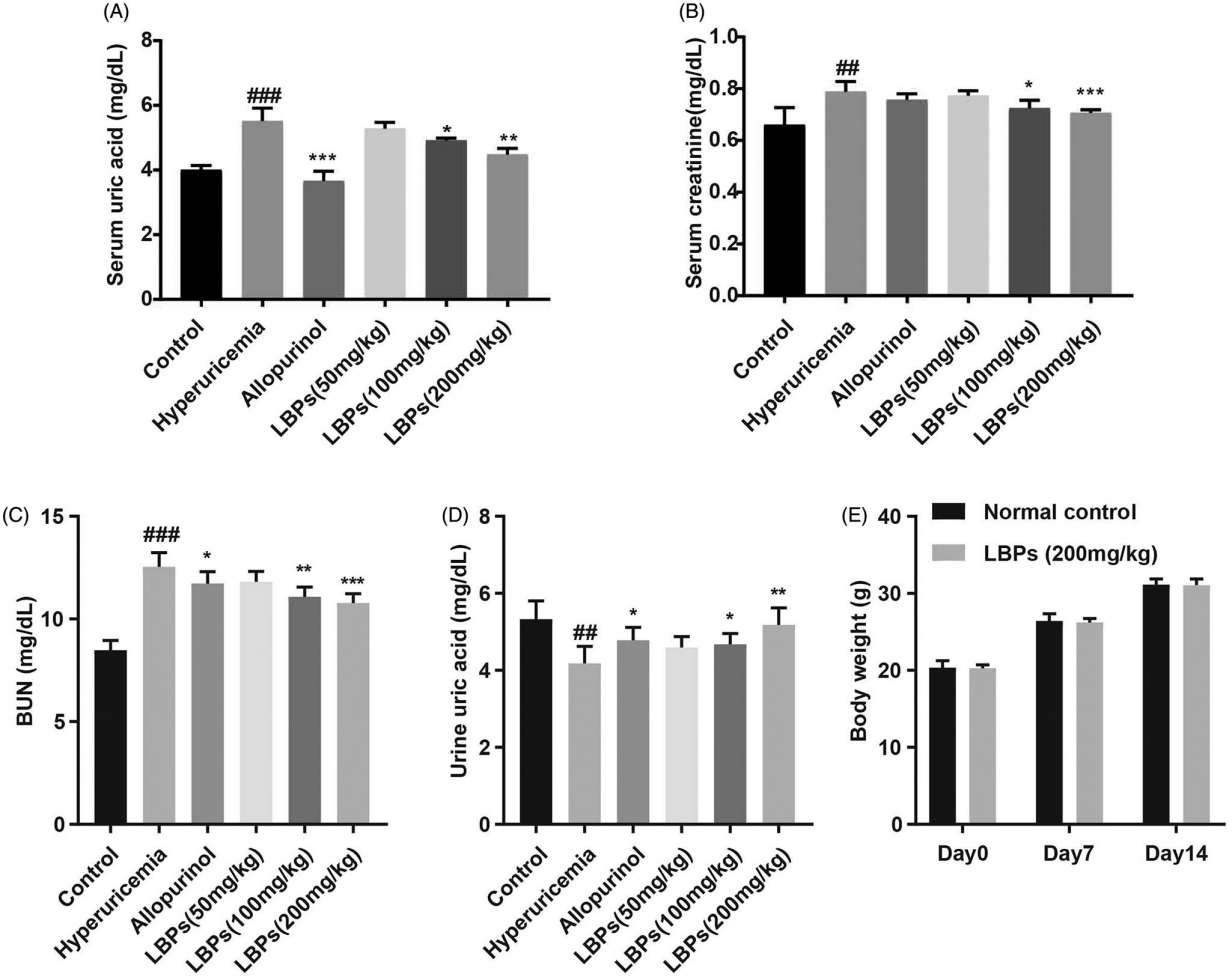
Effect of LBPs on the (A) S_UA_ levels, (B) S_CR_ levels, (C) BUN levels, (D) U_UA_ levels of potassium oxonate-induced hyperuricemic mice and (E) body weight (g) of wild-type C57BL/6 mice. The data are presented as means ± SD, n = 6 per group. ^##^*p* < 0.01 and ^###^*p* < 0.001 compared with the control group. *p < 0.05, **p < 0.01 and ***p < 0.001 compared with the hyperuricaemia group.

After intraperitoneal injection of potassium oxonate, the S_UA_ level was significantly increased in the hyperuricaemia group compared with that in the control group (*p* < 0.001, Figure 1(A)), suggesting that the mouse model of hyperuricaemia was successfully established. The S_UA_ levels in the allopurinol group were reduced compared with those in the hyperuricaemia group (*p* < 0.001, Figure 1(A)), which was used as a positive control. In the LBP (50, 100 and 200 mg/kg) groups, the S_UA_ level was also decreased (*p* < 0.05 or *p* < 0.01) and correlated with the concentration of LBPs, although no significant difference was found between the 50 mg/kg LBP group and hyperuricaemia group, indicating that LBPs reduce the S_UA_ level in the hyperuricemic mice in a dose-dependent manner (Figure 1(A)). At the same time, we examined the levels of S_CR_ and BUN. Consistently, decreases in both the S_CR_ levels and BUN levels were found in the LBP groups (*p* < 0.05, *p* < 0.01 or *p* < 0.001; Figure 1(B,C)). We also examined the U_UA_ levels in all groups. As shown in Figure 1(D), the levels of U_UA_ in the LBP-administered hyperuricemic mice were increased compared with hyperuricemic mice without LPB treatment (*p* < 0.05 or *p* < 0.01), suggesting that LBPs affect the renal excretion of uric acid. What’s more, the acute toxicity study showed no significant changes in body weight (Figure 1(E)), respiration, behaviour changes or death, indicating the safety of the maximal tolerable dose of LBPs. Therefore, LBPs reduced the S_UA_ levels in hyperuricemic mice likely by increasing the excretion of uric acid from the blood into urine.

### Effect of LBPs on the protein and mRNA expression levels of OAT1, OAT3 and GLUT9 in kidney tissue of hyperuricemic mice

**Figure 2.**
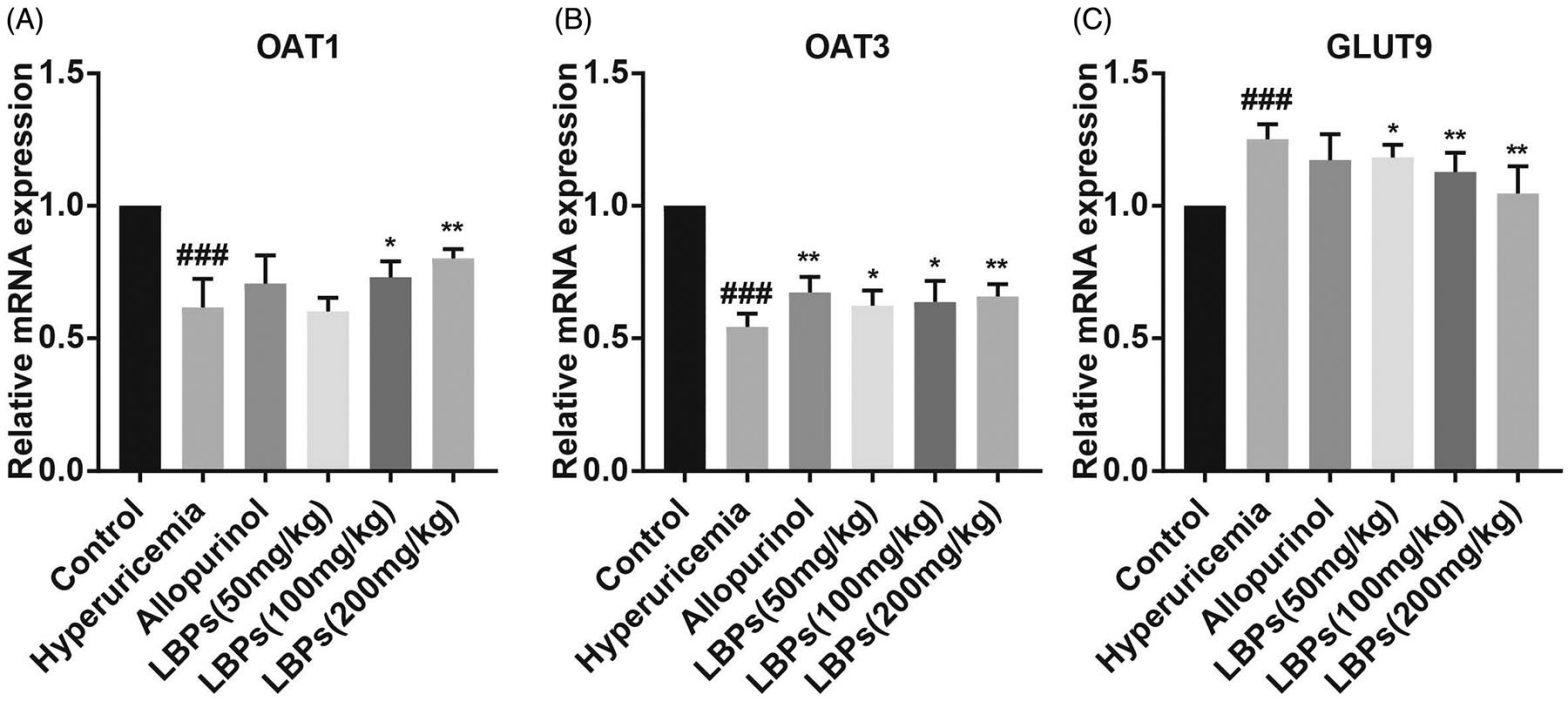
Effect of LBPs on the relative mRNA expression levels of OAT1, OAT3 and GLUT9 in the kidney tissue of potassium oxonate-induced hyperuricemic mice. The data are presented as means ± SD, n = 6 per group. ^###^*p* < 0.001 compared with the control group. *p < 0.05 and **p < 0.01 compared with the hyperuricaemia group.

Many studies have demonstrated that certain molecules are responsible for the excretion and absorption of UA in the kidney, such as OAT1, OAT3 and GLUT9, which are encoded by Slc22a6, Slc22a8 and Slc2a9, respectively. To further investigate the mechanism, we performed quantitative real-time PCR to examine the mRNA levels of OAT1, OAT3 and GLUT9 in mouse kidney tissue in different groups. Compared with the control group, the mRNA levels of OAT1 and OAT3 were downregulated, and that of GLUT9 was upregulated, in the hyperuricaemia group (*p* < 0.001, Figure 2(A–C)), indicating that the transport of UA from the blood into urine was inhibited in hyperuricemic mice. However, LBPs increased the mRNA levels of OAT1 and OAT3 but decreased the mRNA level of GLUT9 in a dose-dependent manner compared with the hyperuricaemia group (*p* < 0.05 or *p* < 0.01, Figure 2(A–C)).

**Figure 3.**
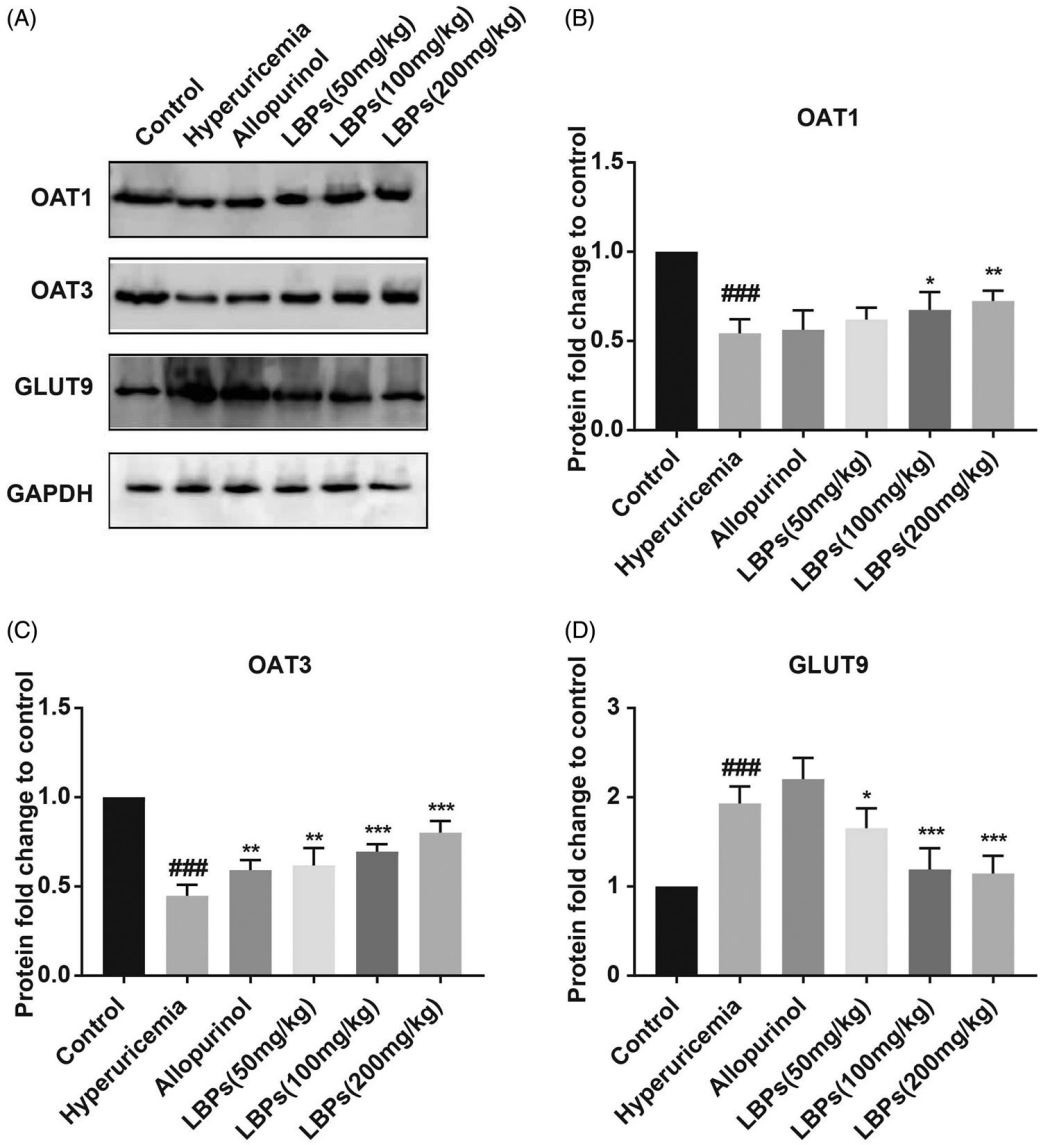
Effect of LBPs on the protein levels of OAT1, OAT3 and GLUT9 in the kidney tissue of potassium oxonate-induced hyperuricemic mice. (A) The protein levels were measured by western blotting, and (B–D) densitometry was calculated. The data are presented as means ± SD, n = 6 per group. ^###^*p* < 0.001 compared with the control group. *p < 0.05, **p < 0.01 and ***p < 0.001 compared with the hyperuricaemia group.

We also detected the protein expression levels of OAT1, OAT3 and GLUT9 by western blot analysis. As shown in Figure 3, although the protein level of OAT3 was elevated in hyperuricemic mice treated with allopurinol (*p* < 0.01), no significant difference was found between the two groups above in the protein expression of OAT1 and GLUT9. However, the protein levels of OAT1 and OAT3 were significantly increased, and that of GLUT9 was decreased, in the LBP groups compared with those in the hyperuricaemia group (*p* < 0.05, *p* < 0.01 or *p* < 0.001; Figure 3). In summary, LBPs affect OAT1, OAT3 and GLUT9 expression, likely by regulating the excretion and absorption of S_UA_ in the hyperuricemic mouse kidney.

### Effect of LBPs on XOD activities in the serum and liver of mice with hyperuricaemia

**Figure 4.**
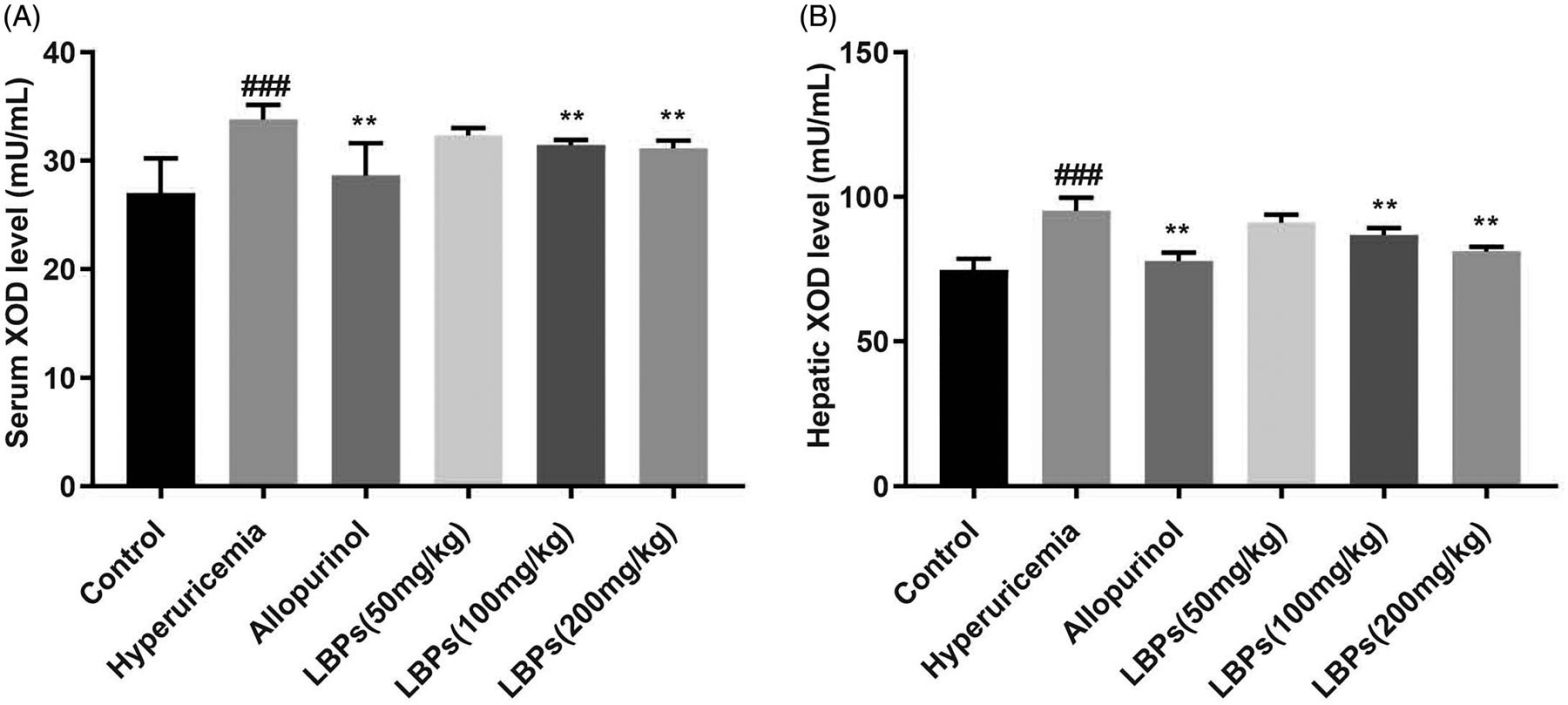
Effect of LBPs on the (A) serum and (B) liver XOD activities of potassium oxonate-induced hyperuricemic mice. The data are presented as means ± SD, n = 6 per group. ^###^*p* < 0.001 compared with the control group. **p < 0.01 compared with the hyperuricaemia group.

We further determined whether LBPs decrease the S_UA_ level in hyperuricemic mice by reducing its synthesis. To test this hypothesis, we tested the serum and hepatic XOD activity in mice. As expected, the serum and hepatic XOD activities were significantly increased in the hyperuricaemia group compared with those in the control group (*p* < 0.001, Figure 4(A,B)); by contrast, the serum XOD and hepatic XOD activities were significantly inhibited in the allopurinol group compared with those in the hyperuricaemia group (*p* < 0.01, Figure 4(A,B)). In the LBP (100 and 200 mg/kg) groups, the serum XOD and hepatic XOD activities were also inhibited compared with those in the hyperuricaemia group (*p* < 0.01, Figure 4(A,B)), indicating the potential mechanism of the decreased S_UA_ levels in hyperuricemic mice treated with LBPs occurs by suppressing XOD activity.

## Discussion

Hyperuricaemia is a metabolic disease caused by disorders of uric acid metabolism, which is characterized by elevated blood uric acid levels. Hyperuricaemia can cause gouty arthritis, renal insufficiency, systemic inflammation, cardiovascular disease, and other metabolic abnormalities (Abeles 2015; Cabău et al. 2020). Hyperuricaemia is mainly caused by insufficient excretion of uric acid by the kidneys or excessive uric acid production by the liver. Uric acid excretion disorders are caused primarily by abnormal urate transporters in the proximal tubules, such as OAT1 (Slc22a6), OAT3 (Slc22a8) and GLUT9 (Slc2a9) (Yong et al. 2017; Fang et al. 2019). Among them, OAT1 and OAT3 are used to excrete urate from the kidney into the urine, which is then excreted from the body, while GLUT9 regulates the reabsorption of urate (Yong et al. 2017; Fang et al. 2019). The excessive production of uric acid is mainly due to the enhanced activity of the key enzyme XOD during uric acid production or the large intake of purines in the diet (Tang et al. 2018). Therefore, regulation of these transporters in the kidney and XOD activity in the liver are key targets for the treatment of hyperuricaemia. Studies have shown that LBPs have protective effects on liver and kidney tissue (Li et al. 2017; Rjeibi et al. 2018; Liao et al. 2019). However, the effect of LBPs on hyperuricaemia remains unclear. In the present study, we observed that LBPs decreased the S_UA_ levels in hyperuricemic mice in a dose-dependent manner. Moreover, the BUN and S_CR_ levels were decreased in hyperuricemic mice treated with LBPs, indicating the protective effect of LBPs on the kidney and corroborating the findings of previous reports (Li et al. 2017; Rjeibi et al. 2017). Conversely, the U_UA_ level was increased in hyperuricemic mice treated with LBPs. These data demonstrated that LBPs reduced the S_UA_ levels, and the underlying mechanism is via increased UA excretion.

To test the hypothesis above, we assessed the mRNA expression levels of Slc22a6, Slc22a8 and Slc2a9 in the kidneys of each mouse group. The results showed that LBPs upregulated the expression levels of Slc22a6 and Slc22a8 but downregulated those of Slc2a9 in the hyperuricemic mouse kidney. At the protein level, LBPs increased the expression levels of OAT1 and OAT3 and decreased the expression level of GLUT9 in the hyperuricemic mouse kidney. These results indicated that LBPs reduce the level of uric acid in the blood by increasing the excretion of uric acid and reducing the reabsorption of uric acid by the kidney. The clinical first-line anti-hyperuricaemia drug benzbromarone works by strengthening the excretion of uric acid in the kidney’s proximal tubule, thereby maintaining blood uric acid at a suitable level. However, many studies have demonstrated the hepatotoxicity of benzbromarone (Wang et al. 2016, 2017), which restricted its usage in some countries, such as the United States.

Allopurinol is another clinical treatment for gout, mainly by inhibiting XOD activity, thereby reducing liver uric acid production. However, studies have reported that allopurinol may cause serious side effects such as rash, liver toxicity or gastrointestinal toxicity (Umamaheswari et al. 2009). Therefore, it is necessary to develop new anti-hyperuricaemia drugs. In the present study, we found that LBPs could significantly reduce XOD activity in the blood and liver of hyperuricemic mice. According to reports in the literature, the pharmacological effects of LBPs are related to their ability to scavenge oxygen free radicals (Li et al. 2007), and active oxygen species, including hydrogen peroxide (H_2_O_2_) and superoxide anion (O^2−^), are produced during the metabolism of purine (Berry and Hare 2004). These findings may explain why LBPs inhibit XOD activity, but further verification is needed.

## Conclusions

Our study demonstrated that LBPs exert an anti-hyperuricaemia effect by increasing the expression of renal OAT1 and OAT3, decreasing the expression of renal GLUT9, and inhibiting XOD activity. The results obtained under our experimental conditions are valuable for the development of new anti-hyperuricaemia agents. Therefore, the anti-hyperuricaemia effect of LBPs on other animals should be further investigated.

## References

[CIT0001] Abeles AM. 2015. Hyperuricemia, gout, and cardiovascular disease: an update. Curr Rheumatol Rep. 17:1936–1949. 10.1007/s11926-015-0495-2 25740704

[CIT0002] Berry CE, Hare JM. 2004. Xanthine oxidoreductase and cardiovascular disease: molecular mechanisms and pathophysiological implications. J Physiol (Lond). 555:589–606. 14694147 10.1113/jphysiol.2003.055913 PMC1664875

[CIT0003] Cabău G, Crișan TO, Klück V, Popp RA, Joosten LAB. 2020. Urate-induced immune programming: consequences for gouty arthritis and hyperuricemia. Immunol Rev. 294:92–105. 31853991 10.1111/imr.12833 PMC7065123

[CIT0004] Cheng J, Zhou ZW, Sheng HP, He LJ, Fan XW, He ZX, Sun T, Zhang X, Zhao RJ, Gu L, et al. 2015. An evidence-based update on the pharmacological activities and possible molecular targets of *Lycium barbarum* polysaccharides. Drug Des Devel Ther. 9:33–78. 10.2147/DDDT.S72892 PMC4277126 25552899

[CIT0005] Choi HK, Mount DB, Reginato AM, American Physiological Society. 2005. Pathogenesis of gout. Ann Intern Med. 143:499–516. 16204163 10.7326/0003-4819-143-7-200510040-00009

[CIT0006] Chung WH, Chang WC, Stocker SL, Juo CG, Graham GG, Lee MH, Williams KM, Tian YC, Juan KC, Jan Wu YJ, et al. 2015. Insights into the poor prognosis of allopurinol-induced severe cutaneous adverse reactions: the impact of renal insufficiency, high plasma levels of oxypurinol and granulysin. Ann Rheum Dis. 74:2157–2164. 25115449 10.1136/annrheumdis-2014-205577

[CIT0007] Fang C, Chen L, He M, Luo Y, Zhou M, Zhang N, Yuan J, Wang H, Xie Y. 2019. Molecular mechanistic insight into the anti-hyperuricemic effect of *Eucommia ulmoides* in mice and rats. Pharm Biol. 57:112–119. 30843748 10.1080/13880209.2019.1568510 PMC6419643

[CIT0008] George J, Struthers AD. 2009. Role of urate, xanthine oxidase and the effects of allopurinol in vascular oxidative stress. Vasc Health Risk Manag. 5:265–272. 19436671 10.2147/vhrm.s4265 PMC2672460

[CIT0009] He L, Tian X, Yan C, Liu D, Wang S, Han Y. 2019. Nicotine promotes the differentiation of C2C12 myoblasts and improves skeletal muscle regeneration in obese mice. Biochem Biophys Res Commun. 511:739–745. 30833077 10.1016/j.bbrc.2019.02.137

[CIT0010] Li XM, Ma YL, Liu XJ. 2007. Effect of the *Lycium barbarum* polysaccharides on age-related oxidative stress in aged mice. J Ethnopharmacol. 111:504–511. 17224253 10.1016/j.jep.2006.12.024

[CIT0011] Li J, Shi M, Ma B, Zheng Y, Niu R, Li K. 2017. Protective effects of fraction 4a of polysaccharides isolated from *Lycium barbarum* against KBrO_3_-induced renal damage in rats. Food Funct. 8:2566–2572. 28671197 10.1039/c6fo01818a

[CIT0012] Liao J, Liu B, Zhong W, Wang GD, Xu YL, Chen X. 2019. Protective effect of *Lycium barbarum* polysaccharides against high-fat diet-induced renal injury and lipid deposition in rat kidneys. J Biol Regul Homeost Agents. 33:7–17. 30666855

[CIT0105] Liu L, Sha XY, Wu YN, Chen MT, Zhong JX. 2020. *Lycium barbarum* polysaccharides protects retinal ganglion cells against oxidative stress injury. Neural Regen Res. 15:1526–1531. 31997818 10.4103/1673-5374.274349 PMC7059572

[CIT0013] Miao Y, Xiao B, Jiang Z, Guo Y, Mao F, Zhao J, Huang X, Guo J. 2010. Growth inhibition and cell-cycle arrest of human gastric cancer cells by *Lycium barbarum* polysaccharide. Med Oncol. 27:785–790. 19669955 10.1007/s12032-009-9286-9

[CIT0014] Po KK, Leung JW, Chan JN, Fung TK, Sanchez-Vidana DI, Sin EL, So KF, Lau BW, Siu AM. 2017. Protective effect of *Lycium barbarum* polysaccharides on dextromethorphan-induced mood impairment and neurogenesis suppression. Brain Res Bull. 134:10–17. 28645861 10.1016/j.brainresbull.2017.06.014

[CIT0015] Pop C, Berce C, Ghibu S, Scurtu I, Sorițău O, Login C, Kiss B, Ștefan MG, Fizeșan I, Silaghi H, et al. 2020. Effects of *Lycium barbarum* L. polysaccharides on inflammation and oxidative stress markers in a pressure overload-induced heart failure rat model. Molecules. 25:466–477. 10.3390/molecules25030466 PMC7037103 31979068

[CIT0016] Rjeibi I, Feriani A, Ben Saad A, Ncib S, Sdayria J, Saidi I, Souid S, Hfaiedh N, Allagui MS. 2017. Phytochemical characterization and bioactivity of *Lycium europaeum*: A focus on antioxidant, antinociceptive, hepatoprotective and nephroprotective effects. Biomed Pharmacother. 95:1441–1450. 28946192 10.1016/j.biopha.2017.09.035

[CIT0017] Rjeibi I, Feriani A, Saad AB, Ncib S, Sdayria J, Hfaiedh N, Allagui MS. 2018. *Lycium europaeum* Linn as a source of polysaccharide with *in vitro* antioxidant activities and *in vivo* anti-inflammatory and hepato-nephroprotective potentials. J Ethnopharmacol. 225:116–127. 29958959 10.1016/j.jep.2018.06.036

[CIT0018] Stavric B, Clayman S, Gadd RE, Hebert D. 1975. Some *in vivo* effects in the rat induced by chlorprothixene and potassium oxonate. Pharmacol Res Commun. 7:117–124. 1144488 10.1016/s0031-6989(75)80015-4

[CIT0019] Tang ZY, Sun D, Qian CW, Chen Q, Duan SS, Sun SY. 2017. *Lycium barbarum* polysaccharide alleviates nonylphenol exposure induced testicular injury in juvenile zebrafish. Int J Biol Macromol. 104:618–623. 28636878 10.1016/j.ijbiomac.2017.06.035

[CIT0020] Tang D, Zhang J, Zhou R, Xie YN, Hou X, Xu K, Wu P. 2018. Phosphorescent inner filter effect-based sensing of xanthine oxidase and its inhibitors with Mn-doped ZnS quantum dots. Nanoscale. 10:8477–8482. 29694472 10.1039/c8nr01355a

[CIT0102] Umamaheswari M, Asokkumar K, Sivashanmugam AT, Remyaraju A, Subhadradevi V, Ravi TK. 2009. *In vitro* xanthine oxidase inhibitory activity of the fractions of Erythrina stricta. Roxb. J Ethnopharmacol. 124:646–648. 19467311 10.1016/j.jep.2009.05.018

[CIT0021] Wang H, Feng Y, Wang Q, Guo X, Huang W, Peng Y, Zheng J. 2016. Cysteine-based protein adduction by epoxide-derived metabolite(s) of benzbromarone. Chem Res Toxicol. 29:2145–2152. 27989145 10.1021/acs.chemrestox.6b00275

[CIT0022] Wang H, Peng Y, Zhang T, Lan Q, Zhao H, Wang W, Zhao Y, Wang X, Pang J, Wang S, et al. 2017. Metabolic epoxidation is a critical step for the development of benzbromarone-induced hepatotoxicity. Drug Metab Dispos. 45:1354–1363. 29021351 10.1124/dmd.117.077818

[CIT0023] Wang K, Wang H, Peng Y, Zheng J. 2016. Identification of epoxide-derived metabolite(s) of benzbromarone. Drug Metab Dispos. 44:607–615. 26792818 10.1124/dmd.115.066803

[CIT0024] Wu HT, He XJ, Hong YK, Ma T, Xu YP, Li HH. 2010. Chemical characterization of *Lycium barbarum* polysaccharides and its inhibition against liver oxidative injury of high-fat mice. Int J Biol Macromol. 46:540–543. 20193709 10.1016/j.ijbiomac.2010.02.010

[CIT0025] Xing X, Liu F, Xiao J, So KF. 2016. Neuro-protective mechanisms of *Lycium barbarum*. Neuromolecular Med. 18:253–263. 27033360 10.1007/s12017-016-8393-y

[CIT0026] Yang CY, Chen CH, Deng ST, Huang CS, Lin YJ, Chen YJ, Wu CY, Hung SI, Chung WH. 2015. Allopurinol use and risk of fatal hypersensitivity reactions: a nationwide population-based study in Taiwan. JAMA Intern Med. 175:1550–1557. 26193384 10.1001/jamainternmed.2015.3536

[CIT0027] Zeng P, Li J, Chen Y, Zhang L. 2019. The structures and biological functions of polysaccharides from traditional Chinese herbs. Prog Mol Biol Transl Sci. 163:423–444. 31030757 10.1016/bs.pmbts.2019.03.003 PMC7102684

[CIT0103] Yong T, Chen S, Xie Y, Chen D, Su J, Shuai O, Jiao C, Zuo D. 2017. Hypouricemic effects of *Ganoderma applanatum* in hyperuricemia mice through OAT1 and GLUT9. Front Pharmacol. 8:996–1007. 29379442 10.3389/fphar.2017.00996 PMC5775298

